# Creativity and Connection: The Impact of inspirED with Secondary School Students

**DOI:** 10.3390/jintelligence11010008

**Published:** 2022-12-31

**Authors:** Jessica D. Hoffmann, Kalee De France, Julie McGarry

**Affiliations:** Yale Center for Emotional Intelligence, Yale University, New Haven, CT 06510, USA

**Keywords:** creativity, emotional intelligence, adolescence, social-emotional learning, school climate

## Abstract

The World Economic Forum predicts that the skills most highly valued by employers in 2025 will be problem-solving, self-management, working with people, and technology use and development. Educators are seeking ways in which to incorporate these skills into their daily instruction. Here, we offer one possible approach to bolster skills in each of these domains: the inspirED program. inspirED was designed for U.S. middle and high schools to support teams of students in completing projects or campaigns that they believe will make their school a better place for all. This study enrolled teams of students from 22 middle and high schools, and provided them with online training, coaching in the inspirED process, and resources to complete their project. Upon finishing their projects, students on the inspirED teams reported higher sense of purpose and self-awareness around the importance of emotions. The larger student bodies at schools in which inspirED projects took place also reported improvements in school climate including students’ perceptions of teaching quality, sense of school pride, student relationships, and emotional safety. Implications and future directions for school-based social-emotional learning and student leadership opportunities are discussed.

## 1. Introduction

In their Future of Jobs Report, the World Economic Forum identifies the top 10 work skills that employees will need in 2025 (Schwab and Zahidi 2020). The list is categorized into four areas: problem-solving, self-management, working with people, and technology use and development. The specific skills listed under these areas include creativity and originality, initiative, stress tolerance, and flexibility, as well as technology use and social influence. The report highlights the need to consider how such skills can be fostered in young people who will soon be entering the workforce. Thus, secondary school educators might ask: in addition to core subject knowledge, how can the necessary skills for creative problem-solving, social and emotional well-being, and healthy use of technology be embedded into teaching?

One solution to this challenge is inspirED, a free program that combines creative problem-solving with social and emotional skills training to support secondary school students in completing a project or campaign meant to better their school community (Hoffmann et al. 2022a; in press). We will first describe the theoretical underpinnings of inspirED and its grounding in a creative problem-solving framework and the theory of emotional intelligence. After an overview of the program itself, we present a quasi-experimental study in which 22 school teams of students and their educator advocates were trained in inspirED and supported through their first projects. In this initial investigation, we explore both individual outcomes of participating team members and school-level outcomes of climate, school satisfaction, daily affective experiences at school, and prosocial intentions.

## 2. Creative Problem-Solving

A creative idea or product is generally defined as one that is both original and useful; however, the idea or the end product alone is only part of the complex process that is creative problem-solving. Thus Plucker et al. (2004) define creativity as “the interaction among aptitude, process, and environment by which an individual or group produces a perceptible product that is both novel and useful as defined within a social context” (p. 90). While many different models of the aforementioned creative process exist, several broad stages are recognized by most: (1) problem construction—the process by which individuals give structure to an ill-defined problem and thereby identify the specific goals of their subsequent problem-solving efforts (Mumford et al. 1994; Reiter-Palmon and Murugavel 2018); (2) idea generation—the act of coming up with multiple solutions to a problem or challenge which increases the likelihood that some of the ideas will be high quality (e.g., Osborn 1953); (3) idea evaluation and execution—the act of determining which idea is the most relevant, effective, and implementable (Dean et al. 2006) and then doing it; and (4) validation—the act of appreciating what one has done and reflecting on the experience as the foundation for launching the next creative act (Shaw 1989, 1994).

Contrary to historical views of creativity as an innate gift, research into the creative process, and specifically the creative problem-solving process, has shown that there are a set of skills people can learn and develop. For example, abundant research shows that the longer a person or team spends on problem construction, the more creative the outcomes (Getzels and Csikszentmihalyi 1975, 1976; Mumford et al. 1997; Reiter-Palmon et al. 1997, 1998). This is a teachable strategy; with guidance, people can be coached to delay problem-solving and spend additional time fully understanding the challenge before moving forward. Moreover, a seemingly endless supply of heuristics exists for stimulating idea generation. The SCAMPER technique (Osborn 1953) for example, encourages people to consider a product or service and then ask questions related to Substituting, Combining, Adapting, Modifying, Putting to another use, Eliminating, and Reversing to generate additional ideas or improvements to the original idea. Finally, even once one has ideas, the selection of the best idea for execution is also a skill (Silvia 2008). For this, guidance can be provided to help people think through the feasibility and relevance of each idea (i.e., Can we complete this in the time allotted? Do we have the right materials? Is this likely to succeed?).

Each of the phases of the creative problem-solving process can also be helped or hindered by emotions, from excitement at a new inspiration to frustration during creative blocks, from insecurity caused by negative critiques to pride in a creative achievement. While much research on creativity and emotion has focused on which emotions enhance or inhibit creativity (e.g., Abele-Brehm 1992; Isen et al. 1985, 1987, 2004), Ivcevic and Hoffmann (2019) introduce a different question: how can any emotion be useful for creativity if a person has the skills to channel it effectively? Their model of emotions in the creative process depicts how each step of the creative process involves emotion and therefore emotion skills, from down-regulating excitement to focus on execution, to summoning the courage to share an idea in class. Indeed, creators across genres concur that channeling and managing their emotions is an integral part of their process (Hoffmann and Russ 2016).

## 3. Emotional Intelligence

How skillfully we use the information our emotions give us is our emotional intelligence, formally defined as “the ability to monitor one’s own and others’ feelings and emotions, to discriminate among them, and to use this information to guide one’s thinking and actions” (Salovey and Mayer 1990, p. 189). This definition further outlines four specific abilities: (1) recognizing emotions in oneself and others, (2) using emotions to facilitate thinking (3) understanding emotions, and (4) regulating emotions. Greater emotional intelligence has been associated with a host of positive outcomes including more effective leadership, satisfaction in social relationships, and academic success (Brackett and Mayer 2003; Kerr et al. 2006; Lopes et al. 2003). Importantly, Brackett et al. (2012, 2019), build upon the model of emotional intelligence, theorizing, and later demonstrating, that emotional intelligence skills can be improved through training and practice when incorporated into school-based social-emotional learning (SEL).

Studies linking the benefits of emotional intelligence skills to creativity are just beginning to emerge. For example, Ivcevic and Brackett (2015) demonstrated the role of emotion regulation ability in bridging the gap between creative potential and creative achievement for adolescents. Other studies suggest that people with higher self-awareness can use emotions (even unwanted ones) to facilitate their creative thinking (Cohen and Andrade 2004; Hoffmann and Russ 2016; Kim et al. 2013), either maintaining a pleasant mood state to facilitate idea generation, or interpreting an unpleasant mood as information to problem find, pivot, or persist in an effort to create a higher quality product.

While both emotional intelligence and creative problem-solving training programs have been established, little work has been done with the two in combination. Moreover, such work has not been fully embedded into the educational context, an opportunity with great potential to reach many young people. The importance of programming that can be implemented within educational settings is emphasized by the ability to impact not only youth who directly participate in the program, but also the larger student body. This is particularly evident when considering the social-emotional elements of the school experience: a study conducted in 2015 found that high school students’ top three feelings at school were tired, stressed, and bored (Moeller et al. 2020). Unfortunately, the COVID-19 pandemic has only exacerbated issues surrounding the social and emotional health of students and the emotional climates of their schools (CDCP 2022).

There is an urgent need for new ideas to address student social-emotional well-being. However, top-down approaches that begin with district offices and adult professional development can take years to reach the students and do not necessarily harness the knowledge, ingenuity, and passion of the young people they are serving. In the current study, we outline a quasi-experimental study designed to investigate the impact and potential of a novel student-led, student-driven, and quickly implementable program that does not require formal educator training called inspirED, that seeks to embed skill-building around both creative problem-solving and emotional intelligence in middle and high school students’ educational experiences.

## 4. inspirED

inspirED is a free, student-led program for middle and high school students. Teams of approximately 4–12 students within a school are provided with emotional intelligence and creative problem-solving training, coaching, and resources to complete a project or campaign at their school that they believe will improve the culture and climate. The inspirED process contains four phases (acronym ABCD): *assess* your school’s climate, *brainstorm* solutions and project ideas, *commit* to and *complete* the project of your choosing, and *debrief* the impact on your community and yourself. Notably, these four phases align with the stages of the creative problem-solving process described previously (i.e., problem construction, idea generation, idea selection and execution, and validation). Students are provided with resources for completing each phase, such as surveys and discussion questions for assessing their climate (e.g., The School Climate Walkthrough Tool; Hoffmann et al. 2022b), empirically supported exercises to foster effective brainstorming (e.g., completing a series of “What if we…” prompts), and motivational tools to encourage persistence, including a “What’s our why?” journaling reflection.

Each inspirED phase is rife with emotions, just as the creative process is more generally. In addition to building creative problem-solving capacity, with guidance and support for students, each phase is replete with opportunities to practice emotional intelligence skills (Hoffmann et al. 2022a; Ivcevic et al. 2022). For example, during the Assess Phase (problem construction), students are asked to examine feelings of frustration, disappointment, or anger to help them notice characteristics, policies, or practices of their school that could be changed or improved (i.e., use their emotions to facilitate thinking and understand the causes of emotions). These skills continue to be practiced when students are in the Brainstorm phase (idea generation), as activities are explicitly designed to foster psychological safety and a sense of team rapport, such that each member is comfortable to share their ideas and think unconventionally. As students complete their project, perhaps the longest phase, they tap into additional emotion skills such as self-regulation, teamwork and communication, and advocacy (recognizing emotions accurately and expressing and regulating emotions effectively).

## 5. The Current Study

The current study was designed to evaluate the inspirED program. The program was delivered to teams of students and educator advocates across 22 middle and high schools. All teams were provided with online training, coaching in the inspirED process, resources to complete their project, and continued guidance from program staff as needed. We specifically hypothesize two sets of results. First, the direct creativity and social-emotional skills training students receive while completing the phases and stages of the inspirED program has been designed to foster long-term growth in how students approach and understand problem-solving and emotions. Therefore, we hypothesize that students who make up the inspirED team will report significant improvements in their creative problem-solving self-efficacy (growth mindset, sense of purpose, and sense of empowerment), as well as their recognition of emotions as information that can support creative thinking, decision-making, and relationships (emotions matter mindset).

Second, inspirED teams choose projects aimed directly at improving one or more areas of the broader school climate. As such, we hypothesize that the effects of the inspirED program are also felt by the larger student body, as measured by changes in school climate. Notably, we specifically hypothesize that the largest school-level changes will be in the domains of school climate that are identified and targeted by inspirED teams. Although exploratory, we also hypothesize that, due to the effects of the inspirED program on the larger school climate, we will find school level increases in school satisfaction, positive affect, prosocial intentions, and decreases in negative affect.

## 6. Method

### Procedure

Schools were recruited through a mailing list newsletter to which they had previously subscribed. Interested schools completed a school agreement form signed by the school leader or designee. A group of 5–11 students were selected at each school to comprise the inspirED team. These students were selected by their schools in a variety of ways. Based on school reporting, 7 teams were drawn from existing student groups including National Honor Society, Positive Behavioral Intervention and Supports team, student council, and student leadership councils and coalitions. Eleven teams were started new, using student recruitment methods such as morning announcements, email blasts, teacher nominations, and student applications. Four teams did not report on their formation process. School teams and the projects they completed are provided in Supplementary Table S2. Selected students turned in informed consent documents and media releases signed by their parents/guardians. One or two educators were assigned by the school leader to be the “educator advocates” and facilitate the inspirED team’s training and project. These educators also completed media releases. Once teams were formed, they were enrolled in the 4-h, asynchronous inspirED online training course.

**Figure 1:**
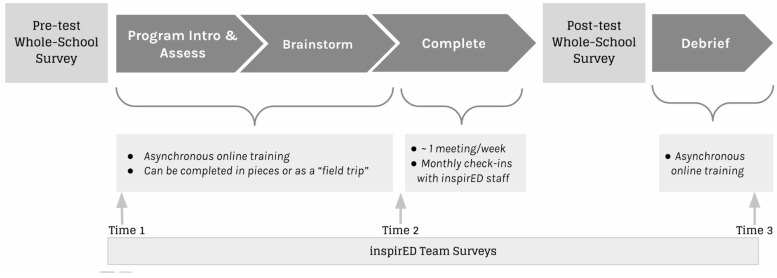
Overview of inspirED Survey and Training Timeline.

Surveys were administered to the entire student body (for those students who assented to participate) at two time points: prior to the inspirED team beginning the training (pre-test) and after the inspirED team completed their project (post-test). inspirED team members completed additional surveys at three time points: prior to training (time 1), immediately following the training and committing to a project idea (time 2), and after project completion (time 3). The entirety of the training and project took place within the course of a single school year (cohort 1: 2020–21 academic year; cohort 2: 2021–22 academic year). An overview of the timeline is depicted in Figure 1.

## 7. The inspirED Intervention

Enrolled school teams received an online training consisting of five modules: Getting Started, Assess, Brainstorm, Complete, and Debrief. Content was presented largely through videos. Teams participated in individual and group reflections, created videos of their ideas and product, and interacted with other teams by posting and responding on discussion boards. The training was flexible and asynchronous, allowing schools to complete it as an “in-house field trip” over one day or in multiple sessions.

The Getting Started module provided an orientation to the training, an introduction to the definition and importance of school climate, and gave a brief overview on prior research related to student experiences at school. The Assess module supported students in discussing and making meaning of their school climate data collected at pre-test, by reviewing a report provided by the research team who had analyzed and aggregated the anonymous responses. The Brainstorm module led students to identify an area of school climate they wanted to target, develop and expand ideas for potential projects, and choose one project to tackle during the study. A priority of the grantor for this study was that teams incorporate peer-to-peer learning with regard to healthy technology use and social media habits, a feature which inspirED teams often choose to incorporate into their projects organically. Here, we explicitly encouraged teams to incorporate an aspect of healthy technology use either as a target of the project itself (e.g., work to reduce cyberbullying in the service of student relationships) or to use technology to advance their project (e.g., incorporate social media posts to spread the word about a campus clean-up day). The Complete module guided students through the project planning and implementation resources available to them including action planning guides, calendars, and skills matrices. Here, teams largely pivoted away from the online modules and into independent planning and working on their project, although they continued to engage with the training platform by logging their meeting notes.

Many schools also took advantage of the offered coaching sessions, where they met with an inspirED coach to check in, receive help navigating roadblocks, or to stay on track and prepare for next steps. Schools also received a research incentive of $350 that they were able to use as they wished. When schools completed their project, they administered the second set of school-wide measures and then returned to the Debrief modules, which prompted teams to analyze their post-test data, identify areas where they had impact, and reflect on their personal growth.

## 8. Participants

This study included 22 middle and high schools in the United States. The schools were a mix of private, traditional public, and alternative, ranging in size from 75 students to 2,826 students, drawn from urban, suburban, and rural locations across 10 states. More information on the characteristics of the schools is provided in Supplementary Table S1.

Across the schools, a total of 15,990 students assented to participate in the study. The sample was 49% female, 48% male, and 3% non-binary/another gender. Students were in grades 6–12; with a majority of middle schoolers—21% 6th graders, 33% 7th graders, 26% 8th graders—and about a fifth of the sample in high school—7% 9th graders, 5% 10th graders, 5% 11th graders, 5% 12th graders. Students reported being between the ages of 11 and 21 (*M*age = 13.00 years, *SD*age = 1.63 years). The racial/ethnic makeup of the sample was: 66% White/Caucasian, 13% Hispanic or Latin, 5% African American or Black, 5% another race, 4% Asian or Asian-American, 3% Biracial or Multiracial, 2% Middle Eastern, and 2% preferring not to answer.

A total of 158 students participated on inspirED teams across the 22 schools. Of those students, 153 assented to and completed research surveys embedded within their inspirED training. Those students who completed surveys were between 11–21 years (*M*age = 13.78 years, *SD*age = 1.91 years), in grades 6–12: 79% middle schoolers, 21% high schoolers. The racial/ethnic makeup was 64% White/Caucasian, 13% Hispanic or Latin, 9% African American or Black, 5% Asian or Asian-American, 5% another race, 2% Biracial or Multiracial, 1% Middle Eastern, and 1% preferring not to answer.

Noting that some students did not complete all survey opportunities, the sample sizes are reported in each results table. Challenges related to COVID-19 school closures and cohorts of students physically attending school on different days led to much of the fluctuation.

## 9. Measures

### 9.1. inspirED Team Measures

The inspirED team members completed measures evaluating their attitude that all emotions matter (e.g., *Emotions are information that help me make good decisions;* 6 items; α = .67), their growth mindset (e.g., *I like projects that challenge me*; 3 items; α = .77), sense of empowerment (e.g., *I feel empowered to make change at my school*; 7 items; α = .90), and sense of purpose (e.g., *I’m discovering things I’m passionate about at school*; 3 items; α = .83). All items were rated on a 1 (strongly disagree) to 4 (strongly agree) scale.

Within the training portal students recorded information on their project ideas, the domains of climate they were aiming to address, the size of their projects, and their answers to reflections on their motivations, challenges, and successes. This information was used to understand the characteristics of students’ projects and to aid with interpretation of findings. Table 1 provides examples of the types of prompts included in the training and sample team responses.

### 9.2. Whole School Measures

All students enrolled in the 22 schools were invited to complete the whole school measures. These included The School Climate Walkthrough (Hoffmann et al. 2022b), a web-based survey application taken in two, 15 min windows on a single school day. Forty-three items are completed in the morning consisting of statements (e.g., *Teachers at my school genuinely care about students*) to which students respond on a 1 (strongly disagree) to 4 (strongly agree) scale. Morning items yield nine domain scores: physical safety (6 items, α = .79), emotional safety (5 items, α = .84), social safety (5 items, α = .81), relationships among students (5 items, α = .81), relationships among adults (4 items, α = .82), relationships between students and adults (5 items, α = .80), teaching quality (7 items, α = .82), respect for diversity (5 items, α = .81), and school pride (6 items, α = .84). The afternoon portion of the scale consists of 72 yes/no statements (e.g., *There were opportunities to get individual help from teachers during class today*). The afternoon items are presented as a percentage of students who endorsed the item and shown on the report under the domains with which they correlate. These item-level results, while not used in pre- and post-test analyses, do help schools develop an action plan by connecting students’ overall school experience with specific school practices.

School Satisfaction was assessed using the school subscale of the Multidimensional Student Life Satisfaction Scale (MSLSS; Huebner 1994). The subscale includes eight statements, reflecting student life satisfaction in the particular domain of school (e.g., *“School is interesting; I wish I didn’t have to go to school;”*), and are rated on a 4-point Likert scale from 1 (*strongly disagree*) to 4 (*strongly agree*). The answers are averaged to form a single school satisfaction score (α = .76).

An adapted version of The Positive and Negative Affect Schedule (PANAS; Watson et al. 1988) was employed. It consisted of 27 feeling words covering both high and low energy pleasant affect (passionate, comfortable), and high and low energy unpleasant affect (angry, bored). Additional emotion words that students frequently report feeling or wanting to feel (e.g., bored, empowered; Moeller et al. 2020) were included. Students were asked how often they felt each feeling word on a scale from 0 to 100% of the time over the past two weeks. Answers are averaged into a score of Positive Affect (17 items, α = .95) and Negative Affect (10 items, α = .89).

The Prosocial Behavior Intentions Scale (Baumsteiger and Siegel 2019), is a 4-item self-report survey that lists prosocial behaviors and asked respondents how likely they are to do those behaviors (e.g., *Help care for a sick friend or relative*). The four items are averaged to form a single prosocial intentions score (α = .68). Prior research with the scale has demonstrated convergent validity with past prosocial behavior and moral identity and predictive validity of future prosocial behavior (Baumsteiger and Siegel 2019).

Finally, at post-test only, students were asked to answer questions about the impact of inspirED. They were first reminded of the inspirED team’s project with a short description supplied by the team that completed the sentence, *“The inspirED team at your school recently completed a project to improve the school’s climate. Specifically,…”*, and asked “Did you know about this?”. For students who answered “yes”, there were four follow-up questions, rated from 1 (strongly disagree) to 4 (strongly agree): (1) This project helped me, (2) I think this project made our school a better place, (3) I hope they do more projects like this in the future, (4) I would like to be part of future projects like this. For students who answered “no” that they had not known of the project, they were asked two follow-up questions: (1) I hope they do more projects like this in the future, and (2) I would like to be part of future projects like this.

## 10. Data Analysis Plan

Driven by our hypotheses, we analyzed our data in two ways. First, we tested the data for improvements in inspirED team members’ reports of emotions matter mindset, sense of empowerment, sense of purpose, and growth mindset. As participants repeated assessments on these measures, we conducted two sets of paired-samples *t*-tests with one-tailed significance testing: the first analysis compared time 1 (pre-program) to time 2 (post-training), and the second compared time 1 (pre-program) to time 3 (post-program completion). Second, we tested the data for significant improvements in domains of school climate. Again, paired-samples *t*-tests were conducted with one-tailed significance testing for each domain of school climate, comparing pre-inspirED program to post-inspirED programming.

## 11. Results

### 11.1. Characteristics of inspirED Projects

The most common domains of climate targeted by projects (teams could select more than one) were relationships among students (6 teams), social safety (6 teams), and emotional safety (5 teams). Student-adult relationships (4 teams), school pride (3 teams), teaching quality (3 teams), and respect for diversity (3 teams) were chosen a moderate amount. The least common domains of climate selected were relationships among the adults (2 teams) and physical safety (2 teams). Most teams chose to incorporate technology by using it to further their school climate project, such as making a video or podcast to spread a message, using online survey tools, or including online meeting spaces for connection. Teams indicated that they felt their projects were medium in difficulty with only a single team reporting their project was easy and one other team indicating their project was difficult. The majority of projects were projected to take “weeks to months” to complete, with just two projects estimated as requiring “days to weeks”, and one project requiring “several months”.

### 11.2. The inspirED Team Outcomes

Paired sample *t*-tests revealed that no significant effects were found from Time 1 (pre-program) to Time 2 (post-training). However, from Time 1 (pre-program) to Time 3 (post-program completion), students did report a significant increase in their sense of purpose and emotions matter mindset, with small effect sizes observed. Sense of empowerment and growth mindset remained unchanged. Results are displayed in Table 2.

### 11.3. School-Level Outcomes

Paired sample *t*-tests conducted at the school level revealed statistically significant increases in four domains of school climate: students’ perceptions of teaching quality, sense of school pride, relationships among students, and emotional safety. Moreover, respect for diversity had an increase of marginal significance. Effect sizes were medium for both teaching quality and emotional safety, and small for school pride, relationships among students and respect for diversity. The remaining domains of school climate did not shift significantly though did show small effect sizes. See Table 3 for school level results.

No significant shifts in school satisfaction, positive affect, negative affect, and prosocial intentions were detected; however, student feedback on the projects themselves was more positive. When asked about the inspirED teams’ work, 66% of students across all the schools indicated that they had known about the project while it was happening. Of those students, 53% agreed the project helped them, 57% agreed the project made their school a better place, 68% hoped the team would do more projects in the future, and 57% wanted to be a part of future projects. For the 33% of students that had not known about the project but were given a description, 65% hoped the team would do more projects in the future, and 42% expressed interest in being involved in future projects.

## 12. Discussion

This study presents preliminary evidence for the inspirED program, a youth driven initiative that aims to bring social-emotional and creativity skills to middle and high school students through the experience of completing a project of their own choosing to improve their school climate. A diverse range of U.S. middle and high schools launched inspirED teams, completed the asynchronous online training, participated in coaching sessions, and successfully completed a project. Projects included launching a peer-to-peer support group, a healthy technology workshop for younger students, a coordinated screen-free day, and a campaign to increase cultural awareness of underrepresented groups.

## 13. Team Member Outcomes

After completing the inspirED program, inspirED team members reported feeling more purposeful and endorsed having more of a mindset that all emotions are useful information than they had held prior to completing the program. While it is not fully possible to determine the mechanisms leading to these shifts within this study, sense of purpose was deliberately targeted due to the Moeller et al. (2020) study indicating purposeful as a feeling students wanted to experience more often. Additionally, we believe that because inspirED allows the students to pick a project about which they are interested and passionate, students may make steps toward feeling as if they have found “their purpose”, whether that be their calling toward a mental health career, a realization that they enjoy advocacy and community organizing, or simply that the change they effect at their school will leave a legacy, giving their time there additional meaning.

This shift to more of an “emotions matter mindset” was also specifically targeted by the inspirED programming. Each step of the ABCD process explicitly indicated the ways in which students might channel their emotions—both pleasant and unpleasant—to their advantage. While this increase was hypothesized, we must recognize that accepting but embracing one’s unpleasant emotions can be difficult. While little work has yet been done on shifting people’s “emotions matter mindsets”, one past intervention with adolescents done by Maliakkal et al. (2016) failed to show a significant change in participants’ attitudes to “emotions matter” items (e.g., unpleasant emotions are a healthy part of life that can help people think creatively), although they were able to shift participants’ growth mindset about creativity and help people recognize the connection between creativity and emotions.

Students’ reported sense of empowerment and growth mindset that students can impact a school’s climate did not show significant changes after inspirED training nor after completion of one semester of the program. One possibility is that merely being put on the inspirED team was empowering and that our first data collection point (i.e., when the team first logged into the inspirED training portal) was not a true baseline. Another possible explanation is the context and timing during which this study ran—the midst of the COVID-19 pandemic, and regardless of inspirED, students may have had other reasons to feel as if their voice was not valued (e.g., masking and vaccine requirements, distance learning).

For the students on the inspirED teams, their projects were an opportunity to practice and build both their social-emotional skills and creative problem-solving skills. Beginning with a hyperlocal project, supported by their educators, students gain a foundational experience on which to build their identity of creative self-efficacy, self-awareness, and prosociality. Based on self-consistency theory (Korman 1970), we can hypothesize that those who think of themselves as prosocial, as problem-solvers, as compassionate, and/or as leaders, are more likely to engage in acts that are consistent with this self-image. In turn, those acts reaffirm the person’s identity. With regard to creativity, research has shown that an increase in creative personal identity (the perception that creativity is an important aspect of the self; Jaussi et al. 2007; Plucker and Makel 2010), is linked to higher creative self-efficacy (a person’s belief that they can solve creative problems when necessary; Bandura 1997; Beghetto 2006) in organizational settings (Tierney and Farmer 2011). With children, however, this self-sustaining cycle must be put in motion; in other words, there must be a first action, success, or experience. For inspirED team members, their project may have served this purpose.

## 14. School Level Outcomes

For the larger student body, students who were the beneficiaries of the school-based projects, significant improvements in perceptions of the school climate following the program’s implementation were noted. Specifically, schools saw, on average, a positive change in relationships among students, emotional safety, school pride, and perceptions of teaching quality. This finding is particularly noteworthy as student relationships and emotional safety were among the most targeted areas of school climate, as were school pride and teaching quality. The impact that students were able to have in a short period of time again highlights the need for more student-led SEL as an effective complement to other recommended adult-led efforts that require time and resources to implement (e.g., smaller class sizes, advisory systems, stronger school-family connections, looping in which students stay with the same teacher over several school years; Darling-Hammond and DePaoli 2020), or miss the mark developmentally regarding adolescents’ need for autonomy (Ozer et al. 2021).

Noteworthy as well were the large percentages of students who indicated that they would like to be involved in future inspirED projects. The pleasant experience of being impacted by an act of kindness or goodness is termed moral elevation (Haidt 2000, 2003), and in addition to inspiring feelings of gratitude, it can also lead to behavior change, such that people will show greater social responsiveness to the needs of others going forward. While hypothesized increases on the prosocial intentions scale were not observed, students’ endorsements that they would like to contribute to future school climate efforts are promising. Much research makes the connection between a more positive school climate and prosocial student behavior and engagement (e.g., Thapa et al. 2013), but this association is likely dynamic and mutually reinforcing. Students attending a school with a positive climate are more likely to engage and behave prosocially, and those same behaviors contribute to a more positive climate. By implementing a school-wide program aimed at improving the school experience for other students, the inspirED program may be able to spark a shift in these dynamics.

Of note, hypothesized changes in school satisfaction and positive and negative affect were not observed. It is possible that school satisfaction, as a more global measure (e.g., I look forward to going to school), would require larger shifts in school climate variables to be detected. Thus, it is also possible that additional projects by the inspirED team would make a difference over time. While the results presented in this study are after completion of teams’ first projects, a consistent culture of student leadership could lead to additional shifts in student experience. With regard to student emotions, natural affective shifts within the school year are due to many factors including holidays, standardized testing, proximity to the summer break, weather, sports seasons, and effects of COVID-19. Thus, while some individual schools noted positive shifts in the emotions reported by the student body, an overall effect due to a single student-led project may be more difficult to achieve.

### Limitations and Future Directions

While this study offers promising findings on the impact of inspirED, it did not include a control group nor random assignment, which are necessary to be sure the effects described were due to inspirED. Large-scale RCTs are an expensive and timely undertaking and require a significant commitment from enrolled schools; thus, it was important to gather preliminary evidence that such an endeavor would be worthwhile. Going forward, such a study might include a longitudinal component to observe the effects of inspirED over several years, and to provide student teams with more time to become established and complete a series of projects. Another limitation is that while the inspirED intervention itself includes the teaching of emotional intelligence skills and creative problem-solving strategies, these skills were not assessed in the present study. Additional measures of emotional intelligence and creative problem-solving in future studies would be beneficial for further demonstrating programmatic impacts and mechanisms.

While we included a diverse set of schools, and sought to have diversity among inspirED team members, this study was too small to analyze research questions related to for whom inspirED works and under what conditions. Creative idea generation likely benefits from a diverse team composed of people with different perspectives, skill sets, and knowledge bases (Amabile 1996; Egan 2005; Hambrick et al. 1996). Future research into inspirED might examine specifically the success of more heterogeneous teams, including teams with students from a range of racial/ethnic backgrounds, students representing various physical, intellectual, and social-emotional abilities, and teams made up of mixed grades and ages. Furthermore, due to the small sample size, we were unable to conduct more rigorous analyses or modeling of how our key variables for inspired team members changed over time. As the program continues, we aim to collect further samples of data and provide more thorough investigations of the impacts of the program.

Finally, while inspirED holds student leadership at its core, school-based programming is not possible without dedicated educators. Future research should examine the specific skills, beliefs, attitudes, and competencies within educator advocates that promote inspirED team success. Likely, some of these skills and attitudes align with the skills the program seeks to develop in students (e.g., a belief that emotions are valuable sources of information) but there are also competencies specific to the educator role. For example, when students discuss aspects of the school that frustrate them, can the educator listen non-defensively while skillfully facilitating their discussion? Is the educator maintaining a sense of intentionality about allowing the process to be driven by the students without exerting undue influence? The constellation of skills and attitudes of the educator can help or hinder the development and experiences of the students on the inspirED team. Further research on inspirED might include specific guidance on the selection of an educator advocate or include additional professional development opportunities.

## 15. Conclusions

The benefits of attending to the social and emotional health of school communities is well-documented (Durlak et al. 2011; Taylor et al. 2017). This includes both the school climate as a whole and the skills of the individual members of the community. At the same time, adolescents are facing a future job market in which the skills of self-management, teamwork, problem-solving, and effective use of technology will be in high demand. inspirED brings these concepts together, putting students in the driver’s seat of making their school a safe, supportive place for all, while building their own skills along the way. Youth around the country and world have shown phenomenal capacity, ability, and desire to take steps to improve their communities and planet, calling to mind examples like Malala Yousafzai, David Hogg, and Greta Thunberg. However, rather than rely on the impact of the few, what if we instead took a broad approach to equip all young people with the support and resources needed to bring their ideas to life? A school-based approach has the power to reach more students, more broadly, and to equip them with the skills that will support them personally, academically, and professionally both now and in the future.

## Figures and Tables

**Table 1:** Sample team responses to inspirED asynchronous training prompts.

Phase	Prompt	Sample Response A	Sample Response B
**Assess**	Healthy technology and school climate: Are there specific issues that come to mind?	“Students being on phones and other devices late at night leading to a lack of sleep that is needed. Students should look to do more positive things when they are using technology.” - Middle School Team, IN	“Not paying attention as much; More distractions at home (remote learning)/staring at a screen; Students posting things that may make others feel uneasy.” - Middle School Team, MI
	School Climate Report Card Discussion	“We agree that tired is a common feeling for high school students. Students get up early and attend school all day and often participate in sports or other activities after school.” - High School Team, MI	“Our most common answer was that the students were feeling bored, happy, and comfortable, because we live in a small town so things don’t change that often which gives a sense of stability but can also get bleak and repetitive.” - Middle School Team, WV
**Brainstorm**	How many ways can you ask yourselves about your school climate? “How can we…”	“How could we help students feel less lonely? What are some reasons that students feel sad? Why aren’t students feeling connected? Why aren’t students seeking help available to them? How can we help students who are feeling angry and discouraged?” - Middle School Team, NM	“How could we create more school community as a whole? How could we encourage motivation and decrease loneliness? Why aren’t there more breaks? What are ways we can take more breaks? What are ways we can get students to feel more hopeful?” - Middle School Team, CO
	Go around so that each person can add a dimension or Idea to this project. “What if we…”	“Assemblies with teachers doing activities: 1. Can make a school video instead of assembly. 2. A game show like Family Feud. Teachers against students. Cohort v cohort. 3. Padlet where teachers post pictures of themselves being ridicu”ous” - Middle School Team, CO	“1. Video to send out to students—what do they do to get rid of the stress 2. Target Mental Health through a video—show examples on how to handle stress 3. Ask for questions and make a video 4. Go into individual seminars to get responses” - Middle School Team, MI
**Commit and Complete**	What’s our “why”? What does our team value and care about?	“Making the school better for everyone who attends, encouraging change, taking advantage of technology to better communicate our messages, positively impacting stud’nts’ lives outside of school by the way they are treated by students and teachers inside of the school.” - High School Team, PA	“We are a group of students who are aiming to improve our school for the better in the long run. We care about our school and the people who attend it and protect it. We are a group of leaders who are trying to make a positive change.” - Middle School Team, WV
	Share your project idea!	“We are going to do a newsletter to let everybody know about games events, attendance awards, birthdays, and other fun things. We will also post on Facebook and online. Our plan [is] to evaluate the progress at the end of the year.” - Middle School Team, MI	“For this project our goal is to have kids feel welcome and safe at our school. We are going to try and achieve this by having students make posters online and send them to us so we can choose the best 30 and hang them up around the school.” - Middle School Team, ND
**Debrief**	Discuss your data	“We were proud that of the students who were aware of our project, most of them were happy about it and thought it made school better. Many of them also said that they would like to be a part of this kind of thing next year. We still want to tackle the student to student relationship piece so that even more kids feel safe and connected at school.” - High School Team, MI	“We were proud that our students felt the following at a high percentage: Positive relationships with adults; Feel physically safe. We were surprised by learning that almost half −48% said they would like to be a part of future projects like”Ie did! Opportunities we would like to tackle are to foster an environment where students feel socially safe because our score was the lowest in this area.” - Middle School Team, PA
	What were your wins?	“Our biggest wins were how much we were able to learn and accomplish during the time given. We were able to work together, each of us taking on different roles, while still having fun… In the end, many students were able to take a screen break during their school day to participate in the hands-on projects….We believe this helped influence a change in mood from students in our school from tired, stressed and bored to happy, hopeful and supported. That to us, is a big win!” - Junior High School Team, KY	“We got to learn new things and see what students think about our school, which was really neat. We got to work with students from different grades that we w’uld not have gotten to know. Even though we were short on time for some of our big ideas, we managed to pull off a project that was positive, noticed, and appreciated by our students and staff.” - Middle School Team, IN

**Table 2:** Changes in inspirED Team Member Outcomes.

Variable	Time 1 vs. Time 2 (*M* Time 1, *M* Time 2)	Effect Size (Cohen’s d)	N	Time 1 vs. Time 3 (*M* Time 1, *M* Time 3)	Effect Size (Cohen’s d)	N
Emotions Matter Mindset	t = −1.32 (3.15, 3.27)	−.21	38	t = −1.93 * (3.15, 3.32)	−.35	31
Sense of Empowerment	t = 1.09 (3.09, 3.00)	.20	38	t = 0.15 (3.04, 3.01)	.03	34
Sense of Purpose	t = −0.18 (3.15, 3.17)	.04	38	t = −1.68 * (3.18, 3.34)	−.31	30
Growth Mindset	t = 0.74 (3.15, 3.09)	.16	38	t = −0.44 (3.04, 3.09)	−.08	34

*Note.* * *p* < .05.

**Table 3:** School-Level Changes in School Climate and Student Affective Experiences.

	Pre-Test *M* (*SD*)	Post-Test *M* (*SD*)	*t*	Effect Size (Cohen’s d)
School Climate Domains				
Teaching Quality	3.08 (0.12)	3.15 (0.17)	−2.71 **	−.58
Physical Safety	3.26 (0.11)	3.28 (0.15)	−1.14	−.24
School Pride	2.98 (0.21)	3.06 (0.24)	−2.17 *	−.46
Respect for Diversity	3.16 (0.14)	3.20 (0.19)	−1.60 °	−.34
Student-Adult Relationships	3.15 (0.14)	3.18 (0.17)	−1.22	−.26
Student-Student Relationships	2.88 (0.16)	2.93 (0.22)	−1.83 *	−.39
Adult-Adult Relationships	3.38 (0.13)	3.41 (0.18)	−.93	−.20
Emotional Safety	2.66 (0.15)	2.75 (0.23)	−2.87 **	−.61
Social Safety	2.80 (0.24)	2.75 (0.13)	1.06	.23
School Satisfaction	2.65 (0.18)	2.64 (0.13)	.28	.06
Positive Affect	58.47 (5.56)	58.91 (5.20)	−.46	−.10
Negative Affect	39.50 (6.73)	38.78 (8.03)	.65	.14
Prosocial Intentions	3.19 (0.12)	3.20 (0.11)	−.36	−.08

*Note*. n = 22 schools. ** *p* < .01, * *p* < .05, ° marginal (*p* = .06).

## Data Availability

The data presented in this study are available on request from the corresponding author.

## Supplementary Materials

The following supporting information can be downloaded at: https://www.mdpi.com/article/10.3390/jintelligence11010008/s1, Table S1. Characteristics of Schools. Table S2. Formation of inspirED teams and their projects.
