# Supplementary material for: Creativity and Connection: The Impact of inspirED with Secondary School Students
Source: J Intell. 2022 Dec 31;11(1):8. doi: 10.3390/jintelligence11010008 (PMC9865255; doi:10.3390/jintelligence11010008)
Supplement: Supplementary file 1 [file jintelligence-11-00008-s001.zip › jintelligence-2000920-Supplementary.pdf]

Journal of
Intelligence

MDPI

Supplementary Table S1. Characteristics of Schools

	School 1	School 2	School 3	School 4	School 5	School 6	School 7	School 8	School 9	School 10	School 11
State	CO	KY	IN	IN	MO	ND	WV	MI	MI	MI	MI
School Size	111	720	653	542	716	75	314	2826	696	1524	1079
Locale	Suburb	City: Small	City: Small	City: Small	Suburb: Large	Town: Distant	Rural: Distant	Suburb: Large	Suburb: Large	Suburb: Large	Suburb: Large
School Type	Alternative	Public	Public	Public	Public	Public	Public	Public	Public	Public	Private
Free/reduced price lunch	64%	56%	56%	62%	36%	19%	63%	17%	19%	13%	Not reported
% Male	62%	52%	51%	54%	49%	56%	48%	51%	50%	48%	Not reported
Grades served	9-12	6-8	7-8	7-8	7-8	6-8	PK-8	9-12	6-8	9-12	PK-8
Race/ethnicity											
African American or Black	7%	22%	19%	18%	8%	5%	0%	8%	1%	3%	3%
American Indian or Alaska Native	1%	1%	0%	0%	1%	0%	0%	0%	0%	0%	0%
Asian or Asian American	0%	4%	2%	1%	0%	3%	0%	3%	1%	43%	1%
Biracial or Multiracial	5%	5%	9%	6%	9%	11%	1%	3%	3%	4%	1%
Hispanic or Latin	34%	19%	30%	51%	17%	13%	0%	4%	9%	3%	1%
Native Hawaiian or Pacific Islander	2%	0%	0%	0%	0%	0%	0%	0%	0%	0%	0%
White or Caucasian	51%	49%	40%	25%	65%	67%	99%	82%	85%	47%	94%

	School 12	School 13	School 14	School 15	School 16	School 17	School 18	School 19	School 20	School 21	School 22**
State	PA	MI	PA	WV	CT	NM	CO	MI	CO	MO	KY
School Size	847	483	1682	733	790	610	1084	1178	473	584	N/A
Locale	Suburb: Large	Rural: Fringe	Suburb: Large	Town: Remote	Suburb: Midsize	Rural: Fringe	Suburb: Large	Suburb: Large	Rural: Fringe	Town: Fringe	Rural: Remote
School Type	Public	Public	Public	Public	Public	Public	Public	Public	Public	Public	Public
Free/reduced price lunch	33%	27%	23%	56%	20%	100%	49%	26%	12%	17%	Not reported
% Male / Female	52%	53%	50%	51%	52%	52%	52%	50%	50%	51%	Not reported
Grades served	7-8	KG-8	9-12	5-8	5-8	7-8	6-8	6-8	6-8	6-7	PK-8
Race/ethnicity											
African American or Black	3%	1%	3%	0%	3%	0%	9%	1%	1%	1%	N/A
American Indian or Alaska Native	0%	0%	0%	0%	1%	0%	1%	0%	0%	0%	N/A
Asian or Asian American	3%	1%	4%	0%	8%	0%	1%	1%	2%	0%	N/A
Biracial or Multiracial	4%	3%	4%	2%	1%	0%	11%	3%	7%	3%	N/A
Hispanic or Latin	10%	2%	9%	0%	14%	97%	27%	9%	9%	4%	N/A
Native Hawaiian or Pacific Islander	0%	0%	0%	0%	0%	0%	0%	0%	0%	0%	N/A
White or Caucasian	79%	93%	81%	97%	74%	3%	49%	86%	81%	91%	N/A

Note. School data is based on the NCES database; **School 22 did not have a report of its 2021-22 student demographics

Supplementary Table S2. Formation of inspirED teams and their projects

**School**	**State**	**New or Existing Group**	**inspirED Project**
High School	CO	Existing	To promote school spirit and inclusivity the team created a community mural where students could take their picture and post on social media with a school hashtag
Junior High	KY	New	To promote student-to-student relationships, teaching quality, and school pride, team created digital survey, raised funds, and designed hands-on learning day to give peers a screen break
Middle School	IN	New	To promote social safety in response to unkindness on social media, created banners at the front entrance and content for morning announcements with messages of encouragement
Middle School	IN	Existing	To promote multiculturalism, researched under-represented cultures and created posters to hang throughout the school
Middle School	MO	Existing	To improve peer-to-peer relationships, organized “Minute to Win It” challenges in advisory classes
Middle School	ND	Existing	To promote social and emotional safety, created a conversation booth at lunch times and invited students to come discuss issues like diversity, technology, self-awareness, and inclusion
PreK-8	WV	Unknown	To promote school pride, created a positive newsletter with school events and updates and promoted through social media
High School	MI	Existing	Wanting to improve relationships between students and students and adults, the team created a peer mentor and mediation group with virtual referral and access options
Middle School	MI	Existing	To normalize struggles with mental health and provide resources, created a video about common sources of stress and ideas to manage stress
High School	MI	New	To combat negativity on social media and high stress levels while promoting bonding, organized monthly fun events, a “get caught being kind” board, and researched the benefits of a “house system” and partnered with administration to implement
PreK-8	MI	New	To support their peers in feeling safe and welcome at school, hosted a graphic design contest to create posters with positive messages for school common areas
Middle School	PA	New	To promote school spirit and give peers a break from screens, planned a screen-free “chill out day” with fun and interactive activities
K-8	MI	Unknown	To address social safety, social skills, and healthy technology use, created educational videos and shared on social media, school newsletter, and school broadcast show to reach students and parents

High School	PA	New	To promote social safety, relationships, and awareness of school happenings, the team created a digital trivia competition with answers sourced from information in the morning announcements
Middle School	WV	Unknown	To improve student-student relationships and promote school pride, the team organized homeroom-based competitions and fundraisers
Middle School	CT	Unknown	To promote more student voice at their school, the team created a suggestion panel made up of students and staff to incorporate students' ideas and feedback
Middle School	NM	New	To support their classmates in managing their emotions and academic demands, the team created and facilitated a virtual peer support group on topics like healthy relationships, mental health, and time management
Middle School	CO	Existing	To boost morale at the school, the team organized and hosted a dodgeball and soccer tournament between advisories and made videos to involve the whole school
Middle School	MI	New	To promote safe behaviors online, the team organized and facilitated a cybersafety workshop for 6th graders
Middle School	CO	New	To increase feelings of motivation at school, the team organized a teacher Jeopardy tournament that they televised during advisories
Middle School	MO	New	To support their peers in promoting mental health, the team did a campaign around healthy mindsets, stress management, and hosted a self-care bingo
PK-8	KY	New	To celebrate diversity at their school, the team organized monthly cultural and heritage events about different cultures and backgrounds
